# Fertility concerns in cancer patients: a bibliometric analysis via CiteSpace: A review

**DOI:** 10.1097/MD.0000000000035211

**Published:** 2023-09-22

**Authors:** Jiali Shen, Hongzhan Jiang, Huihui Lin, Siyue Fan, Doudou Yu, Liping Yang, Lijuan Chen

**Affiliations:** a Nursing College, Fujian University of Traditional Chinese Medicine, Fuzhou, China; b Department of General Surgery, Zhongshan Hospital of Xiamen University, School of Medicine, Xiamen, China.

**Keywords:** bibliometric analysis, cancer, CiteSpace, fertility concerns, Web of Science

## Abstract

Fertility concerns are a pervasive issue but very subtle in patients with cancer. Though various studies have focused on fertility concerns, limited research endeavor has been dedicated to bibliometric analysis. Given this, to visually analyze the hot frontier trends of research related to fertility concerns of patients with cancer using CiteSpace and provide new insights for future research in this field using the bibliometric method. We used CiteSpace software to retrieve the literature related to fertility concerns of patients with cancer in the Web of Science core collection database from the year of establishment to 2022 and conducted visual analysis in terms of authors, countries and regions, research institutions, and keywords. The search resulted in 201 valid articles, and the annual publication volume of literature related to fertility concerns in patients with cancer was generally on the rise; the country with the most publications was the United States, which also had the highest influence; the main research institution was Sloan Kettleson Cancer Research Center; the core research scholar was Jessica R. Gorman; the research hotspots mainly centered on quality of survival, women, survivorship, preservation, breast cancer, adolescence, and infertility. The results of this bibliometric study provide the current status and trends in the fertility concerns of patients with cancer and may help researchers identify the hotspots and frontier trends in this field.

## 1. Introduction

Cancer represents a substantial challenge to global public health, with a high incidence rate, mortality, and medical expenditure.^[1,2]^ According to recent estimates published by GLOBOCAN, approximately 19.3 million newly diagnosed cases and nearly 10 million associated deaths occurred globally in 2020, with projected increases up to 28.4 million cases in 2040, representing a 47% growth.^[3]^ In addition to this sobering rise, oncologic disorders are now affecting progressively younger populations, including those unmarried or childless,^[4–6]^ leading to unique psychological and social distress among these patients, such as reproduction, self/children’s health and raising children issue.^[7,8]^ This is defined as fertility concerns.^[9]^

Many cancer patients express concern over fertility, yet clinicians prioritize physical health over addressing these reproductive issues.^[10]^ Prior research has revealed that many cancer patients are concerned about their future fertility when they are diagnosed, but they do not receive the information they require at that time regarding the risks to fertility and the options for fertility preservation.^[11,12]^ This lack of attention toward reproductive considerations by healthcare providers has been highlighted in earlier studies.^[13]^ Moreover, tackling fertility concerns involves managing cancer patients’ medical and psychosocial aspects.^[14]^ Research on fertility concerns of different cancer patients exists in various fields. It will be hard to enumerate and summarize them individually. This review will systematically sort out and examine the research results of fertility concerns in cancer patients using the method of bibliometric analysis to more thoroughly assess the hotspots and development trends of the study of fertility concerns in cancer patients.

Bibliometric analysis, a technique involving quantitative assessment, has proven useful in exploring trends and offering direction to researchers in multiple domains.^[15]^ Despite its widespread application, there remains limited focus on understanding topical hotspots and developments within the realm of fertility concerns in cancer patients. To our knowledge, no previous efforts have been reported to conduct a bibliometric analysis in this field. Motivated by this gap, we aim to examine fertility concerns in cancer patients through a bibliometric analysis, taking into account country, authorship, and keyword dimensions, thereby providing guidelines for scholars in this area to determine the research direction. By doing so, we hope to contribute meaningfully to advancing the current understanding of the subject matter.

## 2. Methods

### 2.1. Data acquisition

**Figure 1.**
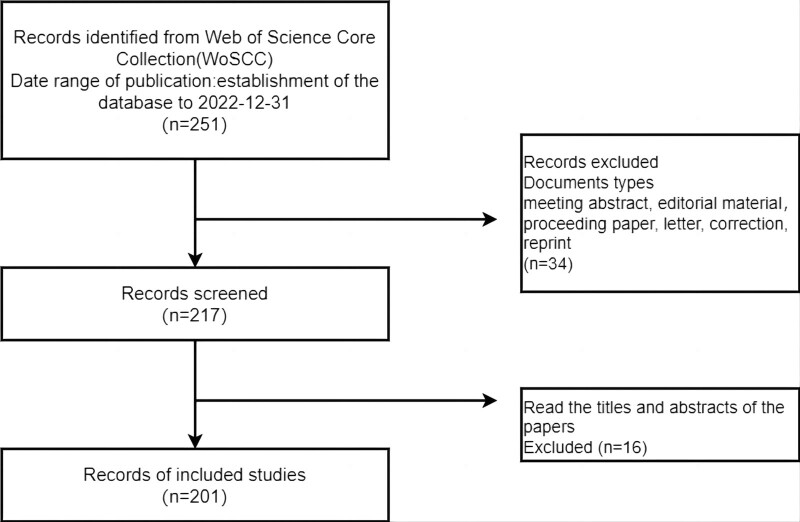
Flowchart of the screening process.

The Web of Science Core Collection database was used as the data source, and the search extended from the establishment of the database to December 31, 2022. Here are the search strategies: 1# TS= ((Fertility concerns) or (Fertility-related concerns) or (reproductive concerns)), 2# TS=((Cancer) or (Tumor) or (Neoplasm) or (Neoplasia) or (Malignant Neoplasm) or (Malignancy) or (Malignancies)). The retrieval strategies are summarized in Table 1. The type of literature was limited to journal articles and reviews. The search was conducted using the Web of Science Core Collection database. Two hundred seventeen papers were identified, and then 2 researchers independently read the titles and abstracts of the papers to exclude those that did not refer to fertility problems related to cancer, and finally, 201 papers were included (Fig. 1).

**Table 1:** Search strategy.

Step	Search strategy	Count
#1	(((((((TS=(“fertility concerns”)) OR TS=(“fertility-related concerns”)) OR TS=(“Reproductive concerns”)) OR TS=(“reproductive-related concerns”)) OR TS=(“fertility worries”)) OR TS=(“fertility anxiety”)) OR TS=(“Reproductive worries”)) OR TS=(“Reproductive anxiety”)	3,958,891
#2	((((((TS = (Tumor)) OR TS = (cancer)) OR TS = (Neoplasm)) OR TS = (Tumors)) OR TS = (Neoplasia)) OR TS = (Neoplasias)) OR TS = (Cancers)	350
#3	#1AND#2	251

Article inclusion criteria: published articles; and articles with research topics related to cancer and fertility concerns. Exclusion criteria: repeated articles; and literature from conferences and newspapers, such as letters, and news reports. The literature that was not relevant to the study and duplicate literature were manually removed by reading the title and abstract. 221 papers were finally included and saved as plain text files in download___xx.txt format after retrieval.

### 2.2. Literature research and analysis method

Employing innovative Citespace (6.2. R2),^[16]^ we analyzed various aspects relating to the authorship, geographical distribution, institutional affiliation, and subject matter associated with the available literature on fertility concerns in cancer patients. These facets were compiled and visually presented in charts to enhance comprehension regarding prominent areas of interest and rising patterns within the field over time. Through this approach, we aimed to facilitate improved understanding and inform future endeavors.

## 3. Results

### 3.1. Annual numbers of publications

**Figure 2.**
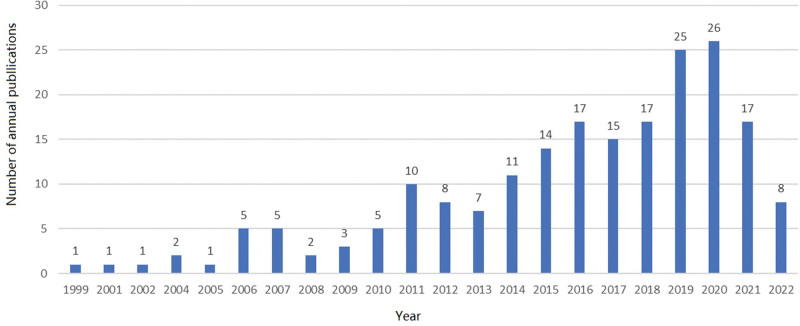
Trend diagram of post volume annual number of articles.

The total number of articles published in the literature related to cancer fertility concerns was 201, and the number of articles published from 1999 to 2010 was in the single digits, with stagnant development; the number of articles published from 2011 to 2022 increased, but the overall development was at a low level; the highest number of articles published was 26 in 2020, with an upward trend in the annual number of articles (Fig. 2).

### 3.2. Analysis of authors

**Figure 3.**
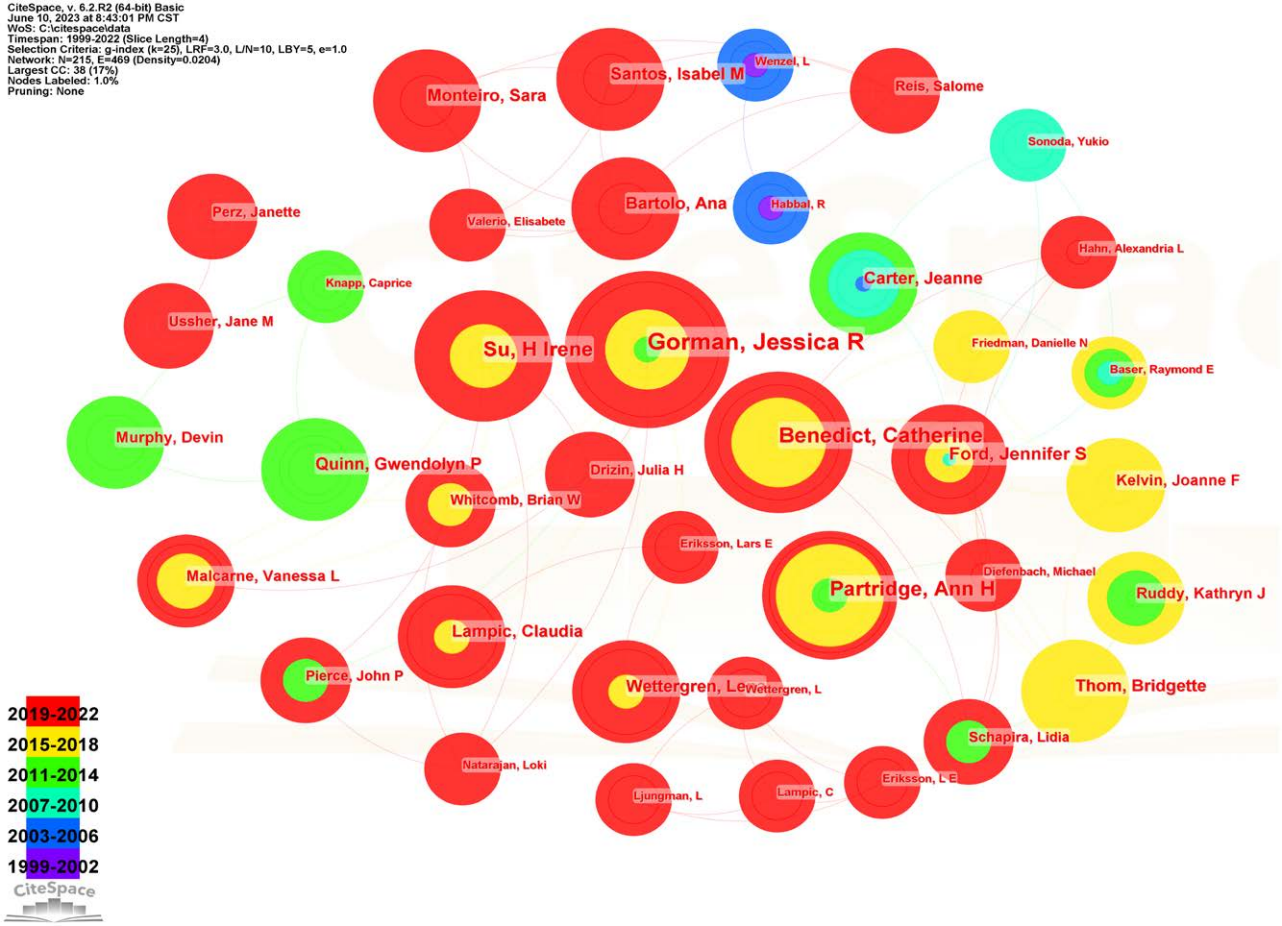
The National Cooperation Map of fertility care research.

Multiple natural clusters can be formed to analyze the authors of articles, reflecting the mainstream groups in the field. Set the parameters in the Citespace function parameter area; the period is 1999 to 2022; the time slice is 4; set the node to the author; click “GO” to run the results; and we can get the authors of articles. The graph (Fig. 3). The graph shows that there are 215 nodes, 469 lines, and a density of 0.0204. Each node represents 1 author, and the number of collaborations between authors is connected; the thicker the line, the more collaborations, and the larger the node, the higher the frequency of appearances. According to statistics, the author with the most articles in this field is Jessica R. Gorman (Table 2). Her 15 articles account for 7.46% of the total number of articles published. This is followed by CATHERINE BENEDICT (11 articles), H IRENE SU (10 articles), ANN H PARTRIDGE (10 articles), JENNIFER S FORD (7 articles), CLAUDIA LAMPIC (6 articles), BRIDGETTE THOM (6 articles), SARA MONTEIRO (6 articles), JEANNE CARTER (6 articles), and ISABEL M SANTOS (6 articles).

**Table 2:** The top 10 authors of the cancer fertility concern study.

Ranking	Author	Count	Year
1	JESSICA R GORMAN	15	2010
2	CATHERINE BENEDICT	11	2016
3	H IRENE SU	10	2015
4	ANN H PARTRIDGE	10	2011
5	JENNIFER S FORD	7	2010
6	CLAUDIA LAMPIC	6	2017
7	BRIDGETTE THOM	6	2016
8	SARA MONTEIRO	6	2020
9	JEANNE CARTER	6	2006
10	ISABEL M SANTOS	6	2020

**Figure 4.**
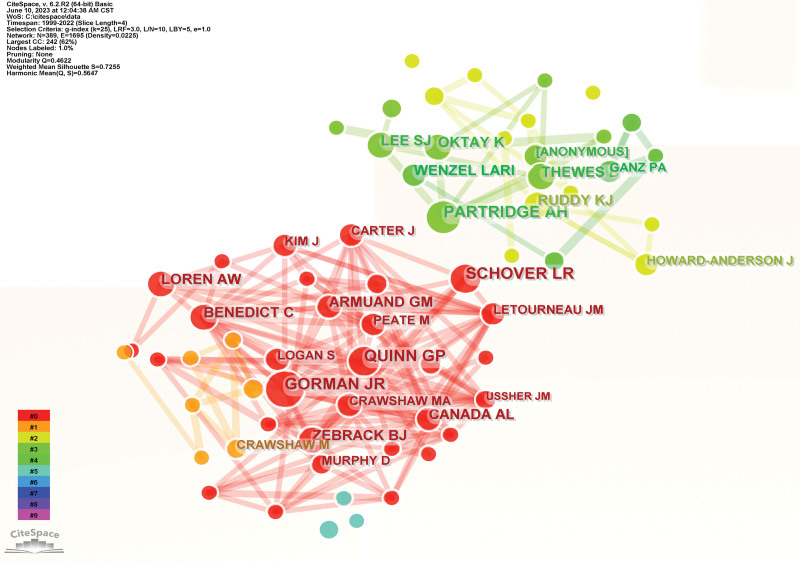
The author co-citation Map of fertility care research.

An author co-citation map was generated that had 389 nodes and 1695 links (Fig. 4). GORMAN JR, PARTRIDGE AH, SCHOVER LR, QUINN GP, RUDDY KJ are the top 10 co-cited authors (Table 3), and the top 3 authors by centrality were SCHOVER LR, QUINN GP, and GORMAN JR. An analysis by centrality and cocitation counts revealed that SCHOVER LR, QUINN GP, and GORMAN JR were core strength researchers.

**Table 3:** Top 10 co-citation in the fertility concern study.

Ranking	Country	Count	Centrality
1	GORMAN JR	84	0.09
2	PARTRIDGE AH	69	0.05
3	SCHOVER LR	60	0.11
4	QUINN GP	59	0.10
5	RUDDY KJ	47	0.05
6	ZEBRACK BJ	45	0.05
7	BENEDICT C	44	0.03
8	LOREN AW	43	0.05
9	LEE SJ	39	0.07
10	OKTAY K	37	0.09

### 3.3. Distribution of countries and institutions

**Figure 5.**
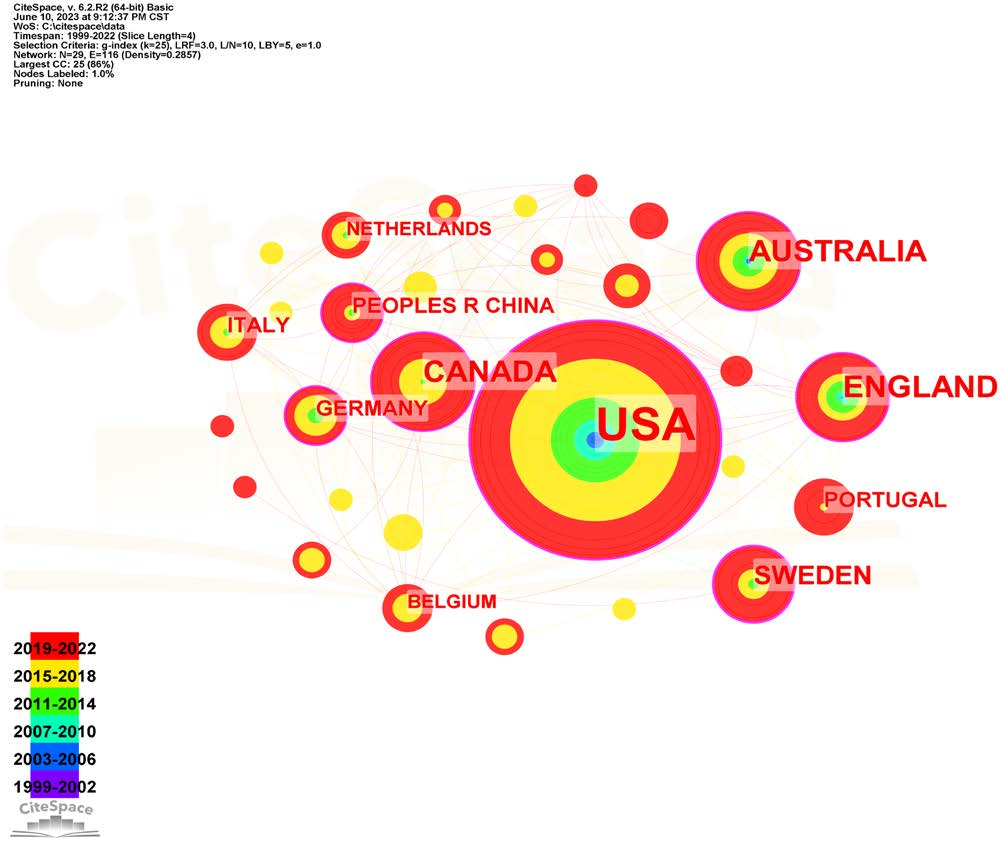
The Cooperative Atlas of the country.

Citespace software can display the number of papers published by countries and research institutions over time in the size and color of “chronology.” Click “GO” to run the result, and a comprehensive analysis of the country or region of cancer patients’ fertility concerns is generated (Fig. 5), with 29 nodes and 116 lines. The larger the nodes, the greater the number of articles issued in that country or region; the more links, the closer the cooperation between countries or regions. The United States has the largest number of publications, among which followed by England (20 articles), Australia (19 articles), Canada (19 articles) Switzerland (12 articles), PEOPLES R CHINA (7 articles), GERMANY (7 articles), PORTUGAL (7 articles), ITALY (6 articles), and NETHERLANDS (5 articles) which shows that the United States has mounted States has the largest number of publications, with 116 articles (57.70%), for in-depth research in the area of fertility concerns (Table 4).

**Table 4:** Top 10 countries in the fertility concern study.

Ranking	Country	Count	Centrality
1	USA	116	0.26
2	ENGLAND	20	0.11
3	AUSTRALIA	19	0.15
4	CANADA	19	0.16
5	SWEDEN	12	0.24
6	PEOPLES R CHINA	7	0.10
7	GERMANY	7	0.10
8	PORTUGAL	7	0.00
9	ITALY	6	0.05
10	NETHERLANDS	5	0.00

The centrality of nodes with circles is greater than 0.1, and the thicker the circle, the greater the centrality, where centrality is an important indicator used to determine the evolutionary process of the discipline and predict the development trend of the discipline, and the higher the centrality indicates the importance of the discipline’s development process.^[17]^ The top 3 countries with higher centrality in this study are the United States (a centrality of 0.26), Sweden (a centrality of 0.24), and Canada (a centrality of 0.16).

**Figure 6.**
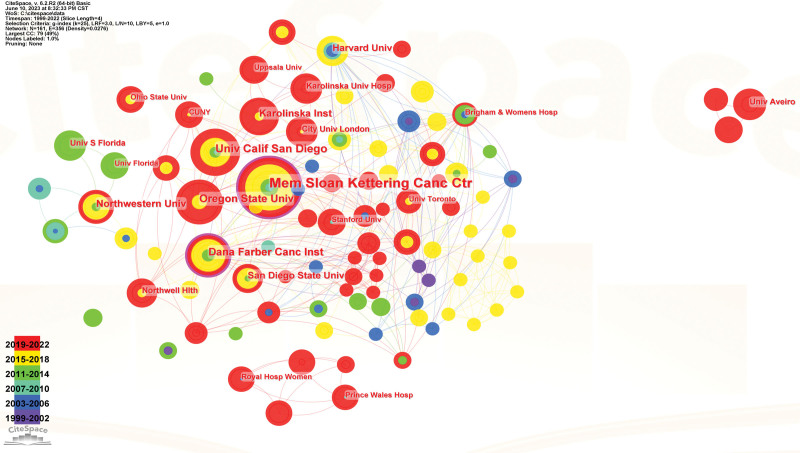
The Cooperative Atlas of the Research Institute.

By analyzing the institutions, we can identify whether there is a cooperative relationship among institutions and find the institutions with more issuing volume by comparing the node sizes in the network graph. The node is set to the institution; other selection strategies remain unchanged; the result is run by clicking “GO,” and the cooperation map of institutions is obtained (Fig. 6). A total of 161 nodes and 356 lines were generated. The larger the nodes, the more the institutions have issued articles; the more links, the stronger the cooperation between institutions. The top 10 institutions in terms of number of articles (Table 5) were Sloan-Kettering Cancer Center (24 articles), University of California, San Diego (13 articles), Oregon State University (12 articles), Dana-Farber Cancer Institute (11 articles), Kellerinska Institute (10 articles), Northwestern Univ (9 articles), Harvard Univ (8 articles), San Diego State Univ (8 articles), City Univ London (6 articles), and Karolinska Univ Hosp (6 articles),

**Table 5:** Top 10 institutions in the fertility concern study.

No.	Institution	Country	Centrality	Count
1	Mem Sloan Kettering Canc Ctr	USA	0.17	24
2	Univ Calif San Diego	USA	0.01	13
3	Oregon State Univ	USA	0.07	12
4	Dana Farber Canc Inst	USA	0.17	11
5	Karolinska Inst	Sweden	0.02	10
6	Northwestern Univ	USA	0.01	9
7	Harvard Univ	USA	0.05	8
8	San Diego State Univ	USA	0.01	8
9	City Univ London	UK	0.00	6
10	Karolinska Univ Hosp	Sweden	0.00	6

### 3.4. Analysis of keywords

**Figure 7.**
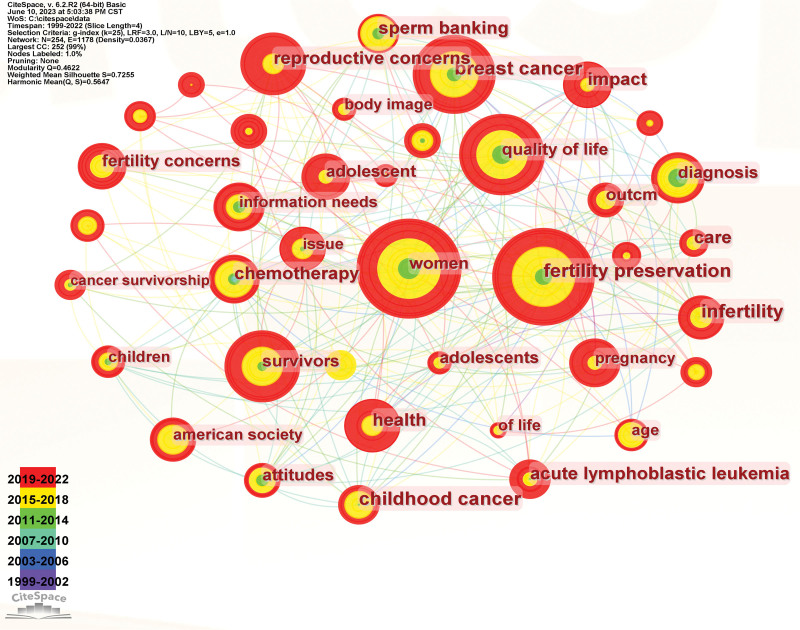
Keyword co-occurrence map.

High-frequency keywords: Keywords are representative words describing the core content of articles, the higher the frequency of occurrence, the higher the research heat, and high-frequency keywords can reflect the hot issues in this research field. Visual analysis of keywords can reflect this field’s research direction, hotspots, and frontiers in various periods (Fig. 7). The top keywords in terms of frequency (Table 6) are “women,” “fertility preservation,” “quality of life,” “breast cancer,” “reproductive concerns,” “survivor,” “health,” “pregnancy,” “childhood cancer,” “impact,” etc.

**Table 6:** Top 10 high-frequency keywords in fertility concerns research.

Ranking	Keywords	Count	Centrality
1	women	93	0.03
2	fertility preservation	91	0.05
3	quality of life	82	0.07
4	breast cancer	72	0.26
5	reproductive concerns	60	0.05
6	survivors	49	0.14
7	health	38	0.11
8	pregnancy	35	0.16
9	childhood cancer	35	0.31
10	impact	34	0.17

**Figure 8.**
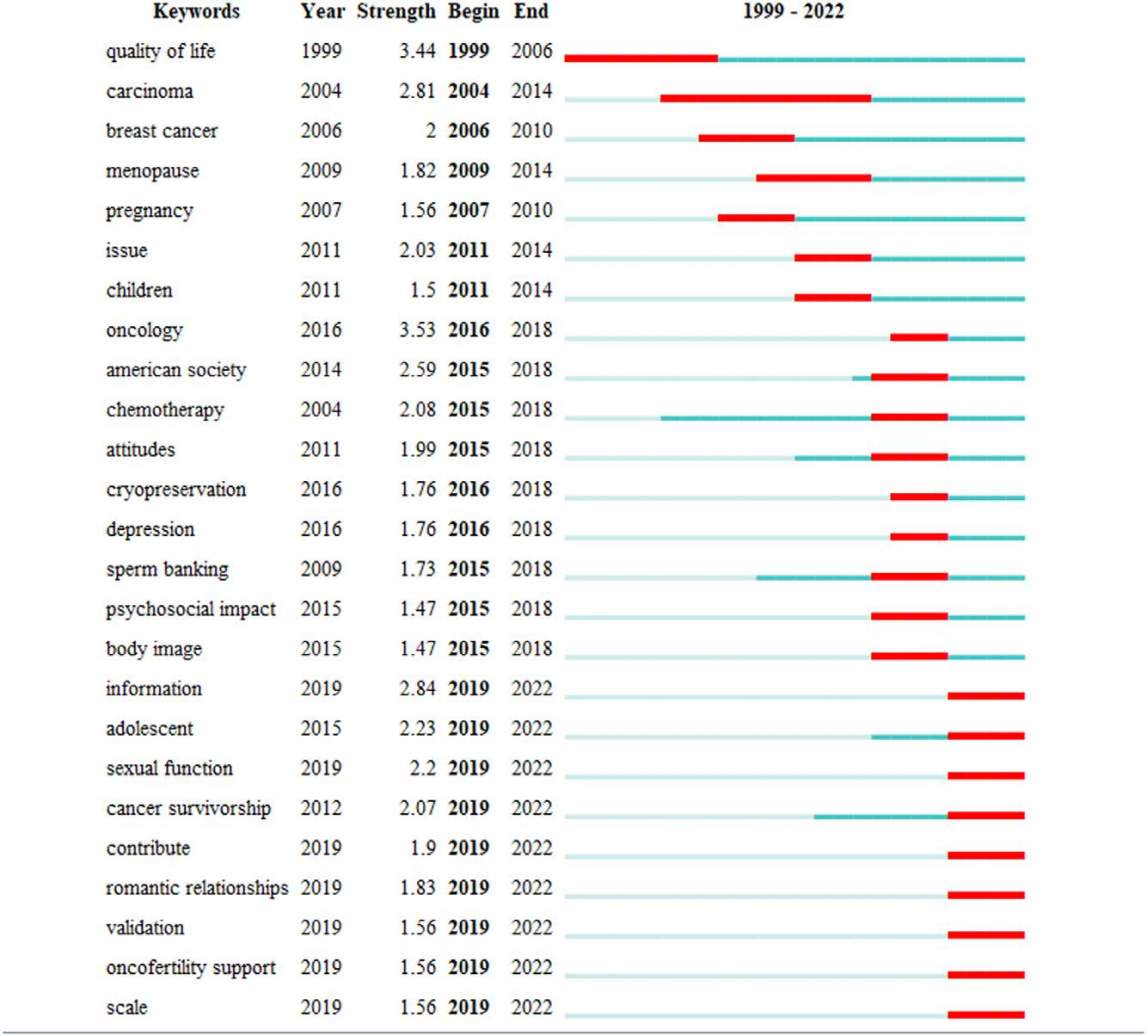
Keywords bursts.

*Keywords bursts*: The emergent keywords refer to the keywords that are used more frequently in a shorter period and can predict the research trends and research frontiers in the field. Reflecting the changes in fertility worry keywords during 1999 to 2022 (Fig. 8). indicate that the research trends can be divided into 3 phases, the first phase in 2015 and before, mainly focusing on “quality of life,” “carcinoma,” “breast cancer,” “menopause,” “pregnancy,” “issue,” “children,” 2015 to 2018 onwards as the second phase, with research including “oncology,” “American society,” “chemotherapy,” “attitudes,” “cryopreservation,” “depression,” “sperm banking,” “psychosocial impact,” “body image” etc. The third phase from 2019 to 2022, with research mainly including “information,” “adolescent,” “sexual function,” “cancer survivorship,” “contribute,” “romantic relationships,” “validation,” “oncofertility support,” etc.

**Figure 9.**
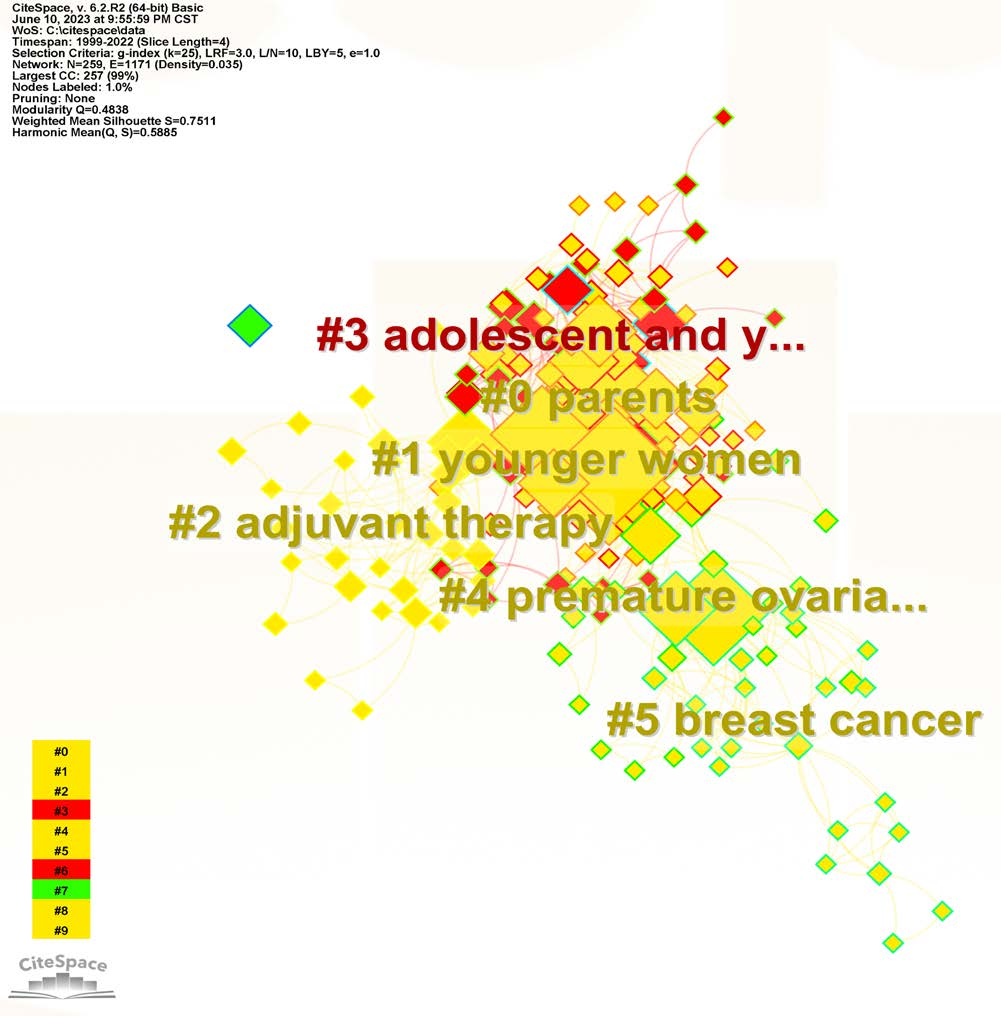
Keyword cluster map.

*Keyword clustering*: Further clustering analysis of keywords in the literature reflects the composition of each research topic in a certain research area, and it was found that the keywords in the literature related to fertility concerns of cancer patients formed 6 clusters (Fig. 9), which are #0 parents, #1 young women, #2 adjust therapy, #3 adolescent and young, #4 premature ovarian failure, #5 breast cancer.

**Figure 10.**
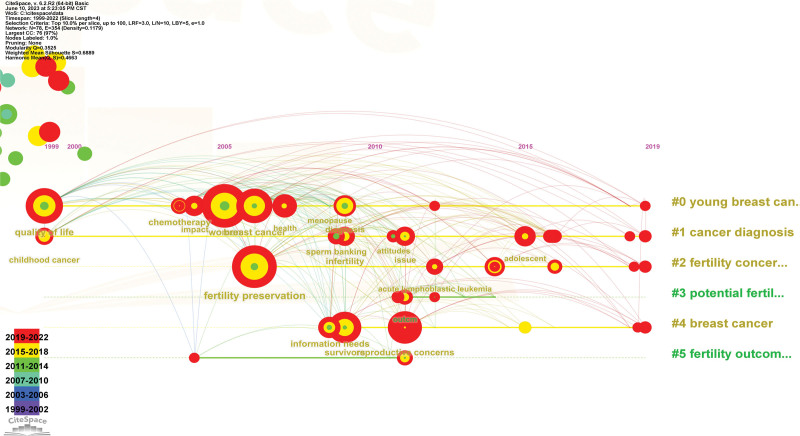
The Horizontal Line of Keyword.

The horizontal line represents the year in which the paper was published, and the vertical line shows different clusters. Each node represents keywords, and the larger the node, the higher the frequency of their occurrence (Fig. 10). It shows that cluster young breast cancer has the longest research period, followed by “cancer diagnosis,” “fertility concerns,” “potential fertility,” “breast cancer” and “fertility outcome” in the latest study.

### 3.5. Analysis of cited references

**Figure 11.**
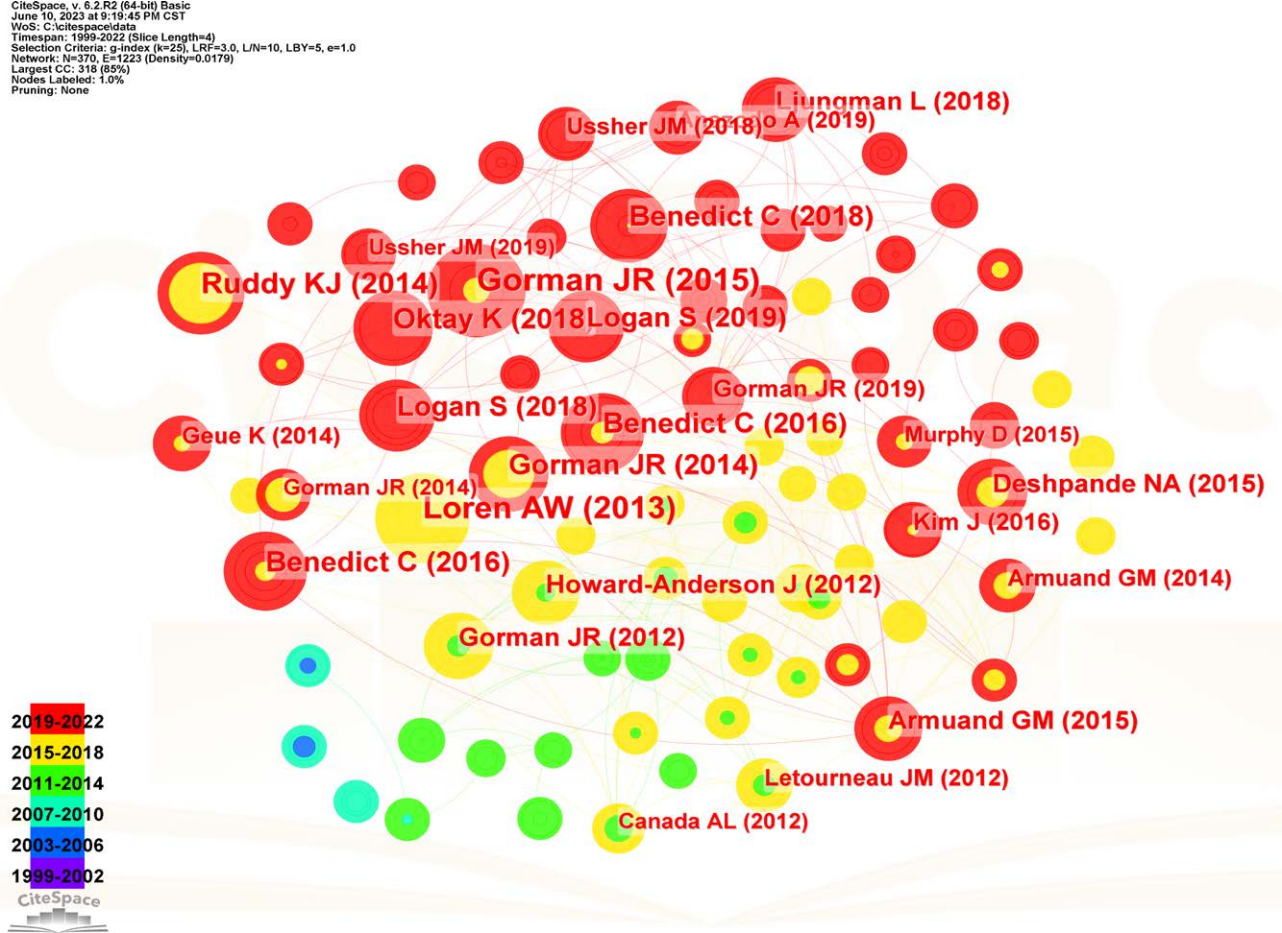
Citation analysis atlas.

The articles were analyzed for citations, and the authors of the top 10 cited articles (Table 7) were, in order: Gorman et al^[18]^ (cited 30 times); Loren et al^[19]^ (cited 28 times); Ruddy et al^[20]^ (cited 23 times); Benedict et al^[21]^ (cited 21 times); Oktay et al^[22]^ (cited 20 times); Gorman et al^[9]^ (cited 20 times); Benedict et al^[23]^ (cited 20 times); Benedict et al^[24]^ (cited 16 times); Logan et al^[25]^ (cited 16 times); and Logan et al^[26]^ (cited 16 times). The high citation rate of the article indicates its high impact in the field (Fig. 11).

**Table 7:** The top 10 cited articles in fertility concerns.

First author	Count	Year	Literature
Gorman JR	30	2015	Experiencing reproductive concerns as a female cancer survivor is associated with depression
Loren AW	28	2013	American Society of Clinical Oncology. Fertility preservation for patients with cancer: American Society of Clinical Oncology clinical practice guideline update
Ruddy KJ	23	2014	Prospective study of fertility concerns and preservation strategies in young women with breast cancer
Benedict C	21	2016	Young adult female cancer survivors’ unmet information needs and reproductive concerns contribute to decisional conflict regarding posttreatment fertility preservation
Oktay K	20	2018	Fertility preservation in patients with cancer: ASCO clinical practice guideline update
Gorman JR	20	2014	A multidimensional scale to measure the reproductive concerns of young adult female cancer survivors
Benedict C	20	2016	Fertility issues in adolescent and young adult cancer survivors
Benedict C	16	2018	Fertility information needs and concerns post-treatment contribute to lowered quality of life among young adult female cancer survivors
Logan S	16	2019	Systematic review of fertility-related psychological distress in cancer patients: informing on an improved model of care
Logan S	16	2018	A systematic review of patient oncofertility support needs in reproductive cancer patients aged 14 to 45 years of age

## 4. Discussion

The research on fertility issues in cancer patients mostly takes place in developed nations, with the USA being home to most research efforts. Three American universities lead the pack when it comes to the number of studies they have published: the University of California, San Diego, Oregon State University, and Memorial Sloan Kettering Cancer Center. American researchers are particularly enthusiastic about studying how cancer affects patients’ chances of conceiving children post-treatment, resulting in America having a greater presence than Europe and Asia across all relevant metrics. Nonetheless, Asian nations, particularly China, South Korea, Japan, and India, contribute significantly in terms of volume to the body of research exploring cancer’s impact on human fertility. While the quality of work varies between research groups, collectively, Asia is establishing itself as a major player in investigations into how best to protect patients’ prospects of starting families later.

Fertility concerns research following a descriptive trajectory remains prevalent throughout the world, chiefly revolving around women afflicted with cancer, although recent years showcase growing interest in addressing similar challenges encountered by males battling malignancy. Studies probing this subject matter often seek to gauge its impact on reproductive health alongside an exploration of contributing factors. Notably, recent studies expose disconcertion harbored by over 60 percent of mature cancer patients who concurrently confront sexual health dilemmas.^[27]^ Further examination into the effects of specific therapies continues apace, including radiotherapy to the abdomen/pelvis, pelvic surgery, alkylating agents, and hormone chemotherapy, amongst other targeted treatments, each implicated in possible side effects on reproduction capacity.^[27,28]^ These works serve to enrich global research on this topic as well as raise awareness regarding fertility preservation options available during the treatment process. A survey by Burgmann et al^[29]^ on 160 young breast cancer patients in Germany showed that 47% of the patients had fertility concerns. American scholars^[30]^ have observed that personal circumstances such as family size and societal ties exert significant influence on fertility concerns. The previous cross-sectional study indicated that educational background, desire for offspring, and preventative steps influence fertility unease.^[31]^ In summary, fertility concerns are influenced by multiple factors.^[32]^

There are few interventions for fertility concerns at present, and the clinical practice guidelines of the American Society of Clinical Oncology strongly suggest that anxiety can be reduced by providing oncology fertility counseling services to patients.^[33]^ According to a randomized controlled trial, using a web-based psychoeducational intervention could improve fertility concerns.^[34]^ In addition, French scholars’ study indicated that fertility preservation methods such as embryo freezing technology, egg freezing technology, and ovarian tissue freezing technology could alleviate the fertility concerns of cancer survivors.^[35]^ In 2006, France included fertility preservation in the law^[36]^ and the American Society of Clinical Oncology has made similar recommendations,^[33]^ but fertility preservation is still controversial in ethics and morality. Overall, there are still relatively few intervention studies on fertility concerns, with small sample size and short intervention time, and its effects need further verification.

Cancer patients often encounter complex issues related to fertility. Consequently, managing such problems requires coordinated input across various professional domains within the framework of the modern biopsychosocial medical paradigm. To address this challenge effectively, multidisciplinary teams should work together, comprising experts from oncology, radiation therapy, reproductive medicine, genetics, and psychological counseling, among others. When devising a comprehensive treatment/fertility plan for patients, professionals should first clarify the individual’s childbearing intentions before implementing a strategy crafted jointly by a collaborative group of specialists aimed at mitigating patients’ fertility fears.^[37]^ Additionally, research suggests that involving multidisciplinary teams improves cancer survivors’ ability to conceive post-therapy and lessens distress associated with infertility concerns.^[38]^

The results of this sciencemetric review will contribute to the research in this field. However, there are some limitations to this study. First, we only included publications in the Web of Science database. Other databases, such as Scopus or PubMed, should be included in the future. Second, the fertility concerns of different cancer patients need to be analyzed separately in the future.

## 5. Conclusion

In summary, our study identified hot topics and frontier trends in the research on fertility concerns among cancer patients through CiteSpace, which may guide new directions for further study. More studies on fertility concerns are needed in the future, including prospective cohort studies and high-quality randomized controlled trials.

## Author contributions

**Conceptualization:** Jiali Shen.

**Data curation:** Hongzhan Jiang, Huihui Lin.

**Formal analysis:** Jiali Shen.

**Methodology:** Hongzhan Jiang, Huihui Lin.

**Software:** Siyue Fan, Doudou Yu, Liping Yang.

**Supervision:** Lijuan Chen.

**Writing – original draft:** Jiali Shen.
